# Age- and Sex-Dependent Changes in Androgen Receptor Expression in the Developing Mouse Cortex and Hippocampus

**DOI:** 10.1155/2015/525369

**Published:** 2015-02-03

**Authors:** Houng-Wei Tsai, Saori Taniguchi, Jason Samoza, Aaron Ridder

**Affiliations:** Department of Biological Sciences, California State University Long Beach, Long Beach, CA 90840, USA

## Abstract

During the perinatal period, male mice are exposed to higher levels of testosterone (T) than females, which promotes sexual dimorphism in their brain structures and behaviors. In addition to acting via estrogen receptors after being locally converted into estradiol by aromatase, T also acts directly through androgen receptor (AR) in the brain. Therefore, we hypothesized that AR expression in the developing mouse cortex and hippocampus was sexually dimorphic. To test our hypothesis, we measured and determined AR mRNA and protein levels in mouse cortex/hippocampus collected on the day of birth (PN0) and 7 (PN7), 14 (PN14), and 21 (PN21) days after birth. We demonstrated that, as age advanced, AR mRNA levels increased in the cortex/hippocampus of both sexes but showed no sex difference. Two AR proteins, the full-length (110 kDa) and a smaller isoform (70 kDa), were detected in the developing mouse cortex/hippocampus with an age-dependent increase in protein levels of both AR isoforms at PN21 and a transient masculine increase in expression of the full-length AR protein on PN7. Thus, we conclude that the postnatal age and sex differences in AR protein expression in combination with the sex differences in circulating T may cause sexual differentiation of the mouse cortex/hippocampus.

## 1. Introduction

Sex differences in brain structure and behavior are ubiquitous in many species, including humans and rodents [1–3]. The gonadal steroid hormones, testosterone (T) and estradiol, are essential for these differences [4–6]. There is a strong link between the sex steroids and gender differences not only in normal physiological functions, but also in the prevalence, symptoms, and associated features of many neurological diseases and mental illnesses [7–10]. For example, the greater the T levels in the amniotic fluid during pregnancy, the higher the child's score on tests of autistic traits [11]. In addition autism is four times more likely to occur in boys than in girls [12]. Therefore, elucidation of the mechanisms underlying the effects of sex steroids on brain sexual differentiation may help identify the essential processes that mediate gender-specific susceptibility to different diseases and assist in the development of new treatments for these sex-biased disorders.

In male mice, the developing testes secrete T with two surges during late gestation (embryonic days 16 and 17, E16 and E17) and on the day of birth (postnatal day 0, PN0), respectively [13–15]. The perinatal rises in T masculinize and/or defeminize the neural circuits underlying sex-specific behaviors [13, 15–17]. Besides reproductive behavior, androgens are important for sex differences in cognitive and social behaviors that are regulated by the cortex and hippocampus [4–6, 18]. In association with distinct behavioral phenotypes between the sexes, there are structural differences in male and female cortex and hippocampus. In adult mice, males possess a thicker cortex than females [19]. During the first week after birth, male rats show a greater rate of neurogenesis in the hippocampus [20]. These differences are associated with the gonadal hormone milieu, especially perinatal T exposure, rather than with the sex chromosome complement. T exerts its effect on brain sexual differentiation by directly binding to androgen receptor (AR) or, after aromatization to estradiol, via estrogen receptors [21–23]. The critical role of T and AR in masculinization of sexually dimorphic brain regions and various behaviors, including cognitive processing, sexual behavior, and response to stress, is supported by previous studies conducted in rodent models lacking functional AR [6, 24, 25].

Analysis of AR expression in the developing and adult rodent brain reveals sexual dimorphism in the bed nucleus of the stria terminalis (BNST) and medial preoptic area (mPOA) with higher AR mRNA and protein levels in males than females [26–28]. In mice and rats, sex differences in AR expression develop in these brain regions during early postnatal development (between PN 4 and 10) [28, 29]. The BNST belongs to the limbic system and has reciprocal connections with the mPOA, and these two brain regions are part of the neural circuitry that controls male sexual behavior in the adult animals [30]. Sexually dimorphic expression of AR in the BNST and mPOA has been implicated to mediate the actions of androgens in the developing brain in between the sexes.

We have previously observed sex differences in histone modifications in the developing mouse cortex/hippocampus with more H3 acetylation on K9/14 (H3K9/14Ac) in males around E18 and PN0 and more H3 trimethylation on K9 (H3K9Me3) in males around PN0 and PN6 [31]. Sex differences in H3K9/14Ac were observed to be regulated by T as a significant increase in H3K9/14Ac but not H3K9Me3 was found in prenatally T-treated female neonates. Since AR interacts with histone modifying enzymes and transcriptional regulation of brain-specific genes by histone modifications has been demonstrated to associate with changes in neural function and behaviors in mice and rats [32–34], our finding suggests that T might act on AR to alter gene expression in the mouse brain around the time of birth.

Based on the importance of T in brain sexual differentiation, we hypothesized that, during early development, AR expression in the mouse cortex/hippocampus might be sexually dimorphic and therefore responsible for the development and function of neural circuits governing distinct behaviors between the sexes. To test our hypothesis, we determined AR mRNA and protein levels by RT-qPCR and immunblotting, respectively. Our results reveal the presence of AR mRNA and two AR protein isoforms (110 kDa and 70 kDa) in the male and female mouse cortex/hippocampus during early postnatal development. While males have higher AR protein expression than females on PN7 only, the developing mouse cortex/hippocampus showed a robust increase in AR mRNA and protein levels as function of age. The developmental profile of AR expression in the mouse cortex/hippocampus not only implicates AR in the organization and function of neural circuits for higher cognitive function, such as learning and memory, but also supports AR as a direct target through which different levels of circulating androgens can differentially regulate cortical and hippocampal development and function between the sexes.

## 2. Materials and Methods

### 2.1. Animals

Adult male and female C57BL/6J (000664) mice were purchased from the Jackson Laboratory (Bar Harbor, ME) and housed in the California State University Long Beach (CSULB) Animal Care Facility. Animals were kept on a 12 : 12 h light : dark cycle (lights on at 0600 h). Food (Teklad Mouse/Rat Diet #7012; Harlan Laboratories, Inc., Indianapolis, IN) and water were provided* ad libitum*. All experimental procedures were approved by CSULB Institutional Animal Care and Use Committee (IACUC) and performed according to Association for Assessment and Accreditation of Laboratory Animal Care International (AAALAC) guidelines.

### 2.2. Experimental Design

Adult female mice were individually paired with a fertile male to produce the pups used in this study. Male and female pups were sacrificed by rapid decapitation on the day of birth (PN0) and 7 (PN7), 14 (PN14), and 21 (PN21) days after birth. These ages were chosen to overlap with the sensitive period for brain masculinization (critical period), during which endogenous or exogenous sex steroids have been shown to be effective at inducing male-like brain structure and function as adult (organizational effect) [35]. The brains were immediately removed from the skulls and coronally blocked rostral to the optic chiasm and caudal to the mammillary body [36]. The top (dorsal) half of the brain block, including the cerebral cortex and hippocampus, was dissected out, frozen on dry ice, and then stored at −80°C until processing for RNA or protein extraction as described previously [31]. Besides the presence (males) or absence (female) of pigmentation in their anogenital regions [37], the gonads and reproductive tracts of all pups were examined to confirm their sexes.

### 2.3. RNA Extraction and cDNA Synthesis

Using RNeasy Lipid Tissue kits (Qiagen Inc., Valencia, CA), total RNA was extracted from the mouse cortex/hippocampus block according to the manufacturer's protocol. Individual samples were diluted (1 : 50) in 10 mM Tris (pH = 7.5) (Sigma-Aldrich, St. Louis, MO) to determine RNA quality and concentration. Absorbance at 260 nm (A_260_), 280 nm (A_280_), and 320 nm (A_320_) was detected by a Bio-Rad SmartSpec Plus spectrophotometer (Hercules, CA). RNA concentration was calculated at A_260_, and purity was determined by the A_260_/A_280_ ratio (between 1.9 and 2.1). The cDNA samples were synthesized from 1 *μ*g extracted RNA in 20-*μ*L reactions using the Bio-Rad iScript cDNA Synthesis Kit. The reverse transcription was performed at 25°C for 5 min, 42°C for 30 min, and 85°C for 5 min in a Bio-Rad MyCycler thermocycler.

### 2.4. Primer Testing for PCR and qPCR

The PCR primers for AR were the same as those described by Bonthuis et al. [38]. The AR primers were checked for sequence specificity using NCBI's Primer-BLAST program (http://www.ncbi.nlm.nih.gov/tools/primer-blast/) against RefSeq RNA. The forward and reverse primers match the sequences of exons 7 and 8 of the mouse AR gene, respectively. In addition, the primers for two house-keeping genes,* Actb* and* Rpl13a*, were also used as described in our previous study [39]. All primers were purchased from Eurofins MWG Operon (Huntsville, AL) and tested in RT-PCR assays for 40 cycles using GoTaq Hot Start Green Master Mix (Promega, Madison, WI) according to the manufacturers' protocol. Initial tests used an annealing temperature of 2°C below the lower melting temperatures of the primers as indicated by Eurofins MWG Operon. PCR products were separated and visualized on a 1.5% agarose gel with ethidium bromide and then visualized by Molecular Imager Gel Doc XR System (Bio-Rad). All primers used in this study produce single PCR products with the correct size in the positive control as calculated and no product in the negative control.

Next, diluted cDNA stocks were prepared with 8 serial dilutions ranging from 1 : 8 to 1 : 1,024. For each qPCR reaction, 4 *μ*L of cDNA, 1 *μ*L of each of forward and reverse primers (840 nM), and 6 *μ*L of Absolute QPCR SYBR Green Mix (Waltham, MA) were mixed and amplified on an Agilent Stratagene MX3000P qPCR system with MxPro QPCR software (Santa Clara, CA) with an initial 15 min denaturation/polymerase-activation at 95°C, 40 cycles of 15 s at 95°C, 1 min at the optimized annealing temperature, and 30 s at 72°C and a dissociation step with 1 min at 95°C, 30 s at 60°C, and 30 s at 95°C. Cycles of threshold (Ct) were analyzed by linear regression with log base 2 of the dilution as the predictor and Ct of the reaction as the response. All of these primers were suitable for quantification with (1) the R2 of the regression of at least 0.98, (2) Ct values at the dilutions of 1 : 4 (AR and* Actb*) and 1 : 16 (*Rpl13a*) below 30, and (3) the dissociation curve with single peaks. The sequences, annealing temperatures used, and sizes of the resulting PCR products were listed in Table 1.

### 2.5. RT-qPCR

RT-qPCR reactions for gene expression quantification were run under the same conditions as described above, with 4 *μ*L of diluted cDNA (1 : 4 for AR and* Actb* and 1 : 16 for* Rpl13a*). Two replicate reactions for each sample were run, and their Ct values averaged before further analysis. For a given sample, unnormalized relative expression values for AR expression were calculated using Pfaffl's method against the PN0 female baseline [40], and the female baseline was established by averaging the Ct values of all PN0 females. Unnormalized relative AR expression values were divided by the geometric mean of the relative expression values of the two reference genes,* Rpl13a* and* Actb*, to yield normalized relative expression values [41]. Values more than two standard deviations from their group mean were trimmed to two standard deviations from the group mean in the same direction as described previously [39]. In addition, relative* Xist* mRNA levels were similarly measured to further confirm the sexes of the pups used.

### 2.6. Protein Extraction

Mouse brain tissues, including the cortex and hippocampus, were homogenized in cold modified T-PER buffer (Pierce Biotechnology, Rockford, IL) with protease inhibitor cocktail (Sigma-Aldrich) and PMSF (1 mM; Sigma-Aldrich). Brain tissues were homogenized by drawing and ejecting 15–20 times through sterile 20 G needles, followed by centrifugation at 10,000 g for 5 min at 4°C. The supernatant was separated and stored at −80°C. The lysate protein concentrations of individual samples were determined by bicinchoninic acid (BCA) assays (Pierce Biotechnology) before electrophoresis.

### 2.7. Immunoblotting

Since the protein samples from eight groups of mice (*n* = 6-7 per group) could not be run together on the same gel, immunoblotting was conducted by comparing two different sexes (females versus males) at each age (PN0, PN7, PN14, or PN21) or two different ages (PN0 versus PN21) of the same sex (males or females). Protein samples of 20 *μ*g each were first separated on 8% sodium dodecyl sulfate polyacrylamide gel electrophoresis (SDS-PAGE) gels or Any kD Mini-PROTEAN TGX precast gels (BIO-Rad) and then transferred to nitrocellulose membranes using the BioRad Trans-Blot Turbo Transfer System. Each membrane was rinsed then blocked in Tris-buffered saline with 0.1% Tween 20 (TBST) containing 10% milk at 4°C overnight. After blocking, blots were rinsed with TBST and then incubated with the primary antibody against AR (1 : 5,000; N-20; Santa Cruz Biotechnology, Inc., Dallas, TX) for 1 h at room temperature. After rinsing, blots were incubated for 1 h in a horseradish peroxidase- (HRP-) conjugated, anti-rabbit IgG secondary antibody (1 : 10,000; Sigma-Aldrich), followed by detection of AR protein bands with SuperSignal West Pico Chemiluminescent Substrate (Pierce Biotechnology) using the ProteinSimple FluorChem E System (Santa Clara, CA). The blots were reprobed with the antibody against *β*-actin (1 : 200,000; Sigma-Aldrich) with the anti-mouse IgG secondary antibody (Sigma-Aldrich). The intensities of AR and *β*-actin on individual membranes were measured by densitometry and analyzed with AlphaView (ProteinSimple). To calculate relative AR protein levels of individual samples, the densities of AR were first normalized with the densities of *β*-actin, and the ratios of AR and *β*-actin levels in individual samples were then standardized to the mean level of females at the same developmental stage (sex effect) or the mean level of the PN0 group of the same sex (age effect). Each sample was expressed as the fold-difference from either the female or PN0 value, which were both set at 1-fold.

### 2.8. Statistical Analyses

The body weight, tissue weight, and RT-qPCR data from individual groups were first tested for normality of the residuals by the Anderson-Darling test and next tested for equality of variances using Levene's test. If significant deviations were detected (*P* < 0.05), a log transformation was applied to the data. After showing normality and homogeneity of variances, the data were analyzed by two-way ANOVA to evaluate the effects of sex, age, and their interaction. When two-way ANOVA showed significant differences, Tukey's post hoc test was used to determine which groups were significantly different. A  *P* value <0.05 was considered statistically significant.

Relative AR protein data were analyzed using Student's *t*-tests with either sex or age as the single factor. The ratios of the smaller AR isoform (70 kDa) to the full-length (110 kDa) were similarly analyzed with two-way ANOVA to reveal the effects of sex and age and their interactions. A  *P* value of <0.05 was considered statistically significant.

## 3. Results

### 3.1. Body Weight and Brain Tissue Weight of Mice

The numbers of mice at different ages and sexes used in the current study and their body weights and cortex/hippocampus weights are listed in Table 2. No significant sex difference in mean body weight (*P* = 0.506) or tissue weight (*P* = 0.55) was observed (Table 2). In contrast, there was a significant effect of age on body weight and brain tissue weight (*P* < 0.001), the minimum occurring on PN0 and the maximum on PN21. Interestingly, while the body weights of these animals continued to increase as age advanced, the maximum brain tissue weight was attained by PN14.

### 3.2. AR mRNA Levels in the Developing Mouse Cortex/Hippocampus

**Figure 1:**
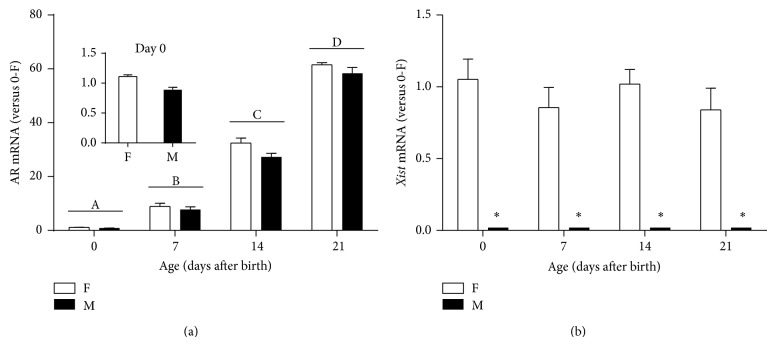
Relative androgen receptor (AR) and* Xist *mRNA levels in the developing female (F) and male (M) mouse cortex/hippocampus measured by RT-qPCR. AR (a) and* Xist* (b) expression was calculated relative to the average in neonatal females (0-F). Different letters indicate significant differences between two ages (*P* < 0.05). ^*^Indicates significant difference between the sexes (*P* < 0.05). Bars are mean ± SEM.

We used RT-qPCR to determine relative mRNA levels of AR in the mouse cortex/hippocampus on PN0, PN7, PN14, and PN21. A two-way ANOVA revealed a significant effect of age (*P* < 0.001) but not sex (*P* = 0.058) or their interaction (*P* = 0.567) on AR mRNA expression. Using Tukey's post hoc test, we further demonstrated that AR mRNA levels in the cortex/hippocampus were significantly higher on PN7 (8.29 ± 0.77, *n* = 17) than PN0 (1.06 ± 0.05, *n* = 36) and continued to rise on PN14 (29.89 ± 1.29, *n* = 16) and PN21 (59.10 ± 1.36, *n* = 16) (Figure 1(a)).

Although the sex difference did not reach statistical significance (*P* = 0.058), there was a trend toward a female bias in AR expression in the mouse cortex/hippocampus, especially on PN14 (females, 32.45 ± 1.88, *n* = 8 versus males, 27.33 ± 1.32, *n* = 8). In addition, relative* Xist* mRNA levels were measured in these animals, and this X-linked gene was exclusively expressed in all female pups, not males, regardless of age (Figure 1(b)).

### 3.3. Detection of AR Protein in the Mouse Cortex/Hippocampus and Hypothalamus

**Figure 2:**
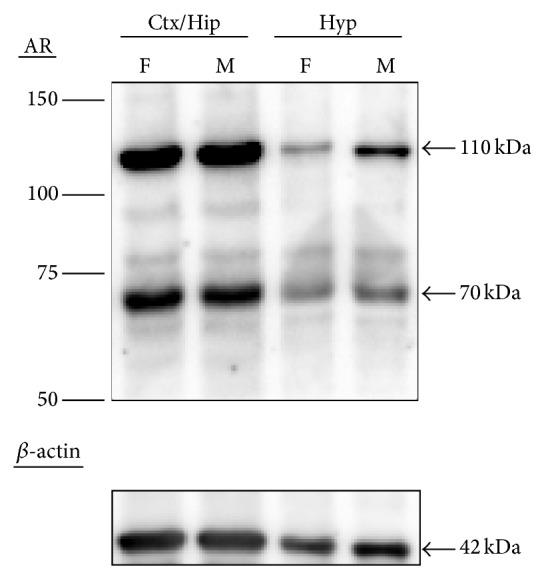
Representative immunoblotting of androgen receptor (AR) protein in the adult female (F) and male (M) mouse brains. Full-length AR (110 kDa) and smaller (70 kDa) isoform as well as *β*-actin (42 kDa) were detected in the cortex/hippocampus (Ctx/Hip) and hypothalamus (Hyp).

Immunoblotting with the antibody against the aminoterminal of AR protein was used to detect AR proteins in the adult mouse brain. As shown in Figure 2, two AR protein bands were observed in the adult mouse cortex/hippocampus and hypothalamus, the full-length AR with a molecular weight of 110 kDa and a smaller isoform of approximately 70 kDa. In the representative blot, the mouse hypothalamus showed a male-biased expression of AR proteins (full-length: female, 1.00 versus male, 2.69-fold; smaller isoform: female, 1.00 versus male, 3.10-fold). In contrast, in the cortex/hippocampus, the AR protein levels were similar between the sexes (full-length: female, 1.00 versus male, 1.04-fold; smaller isoform: female, 1.00 versus male, 0.86-fold).

### 3.4. Effect of Sex on AR Protein Levels in the Developing Cortex/Hippocampus

**Figure 3:**
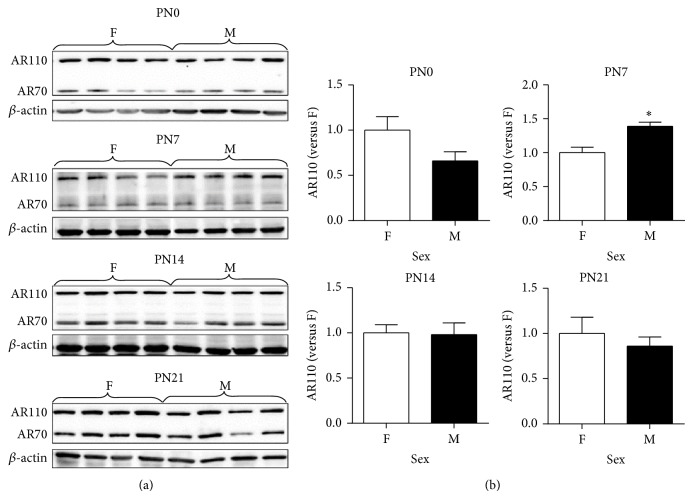
Effect of sex on expression of the full-length androgen receptor (AR) protein (AR110) and lower molecular weight isoform (AR70) in the developing female (F, *n* = 5-6) and male (M, *n* = 6-7) mouse cortex/hippocampus measured by immunoblotting. (a) Representative immunoblotting of AR110 and AR70 as well as *β*-actin in the mouse cortex/hippocampus collected on postnatal days 0 (PN0), 7 (PN7), 14 (PN14), and 21 (PN21). (b) Relative AR110 levels were quantified relative to average in females of the same age. ^*^Indicates significant difference from female expression by *t*-test (*P* < 0.05). Bars are mean ± SEM. PN, postnatal day.

We first tested if AR protein expression in the developing mouse cortex/hippocampus was different between the sexes at the protein level. Because all samples could not be run on the same gel, we performed immunoblotting with same-age male and female samples on a single blot to test the effect of sex on AR protein expression. Representative immunoblots were shown in Figure 3(a). Relative AR levels of individual samples were calculated by normalizing to the mean ratio of AR versus *β*-actin of females in the same developmental stage. A *t*-test was conducted to evaluate the effect of sex on AR protein expression in the mouse cortex/hippocampus at PN0, PN7, PN14, and PN21. As shown in Figure 3(b), on PN0, full-length AR protein levels in males (0.66 ± 0.10, *n* = 7) were 66% of those in female controls (1.01 ± 0.15, *n* = 6) (*P* = 0.084); however, this trend was reversed on PN7 when AR protein levels were 1.3 times greater in males (1.39 ± 0.06, *n* = 6) than in females (1.00 ± 0.08, *n* = 6) (*P* = 0.004). On PN14 and PN21, this sex difference in AR protein levels disappeared. Unlike the full-length AR, there was no sex difference in levels of the smaller AR isoform at any time (PN0, *P* = 0.407; PN7, *P* = 0.603; PN14, *P* = 0.670; and PN21, *P* = 0.758) (Table 3).

### 3.5. Age-Dependent Increase in AR Protein of the Developing Cortex/Hippocampus

**Figure 4:**
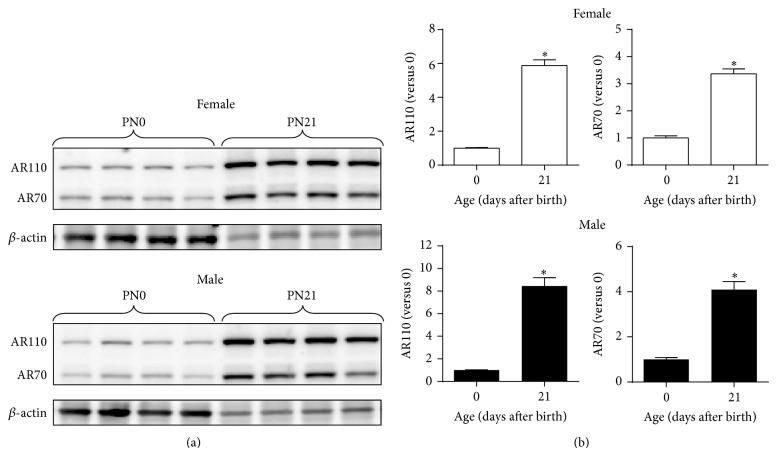
Effect of age on expression of androgen receptor (AR) proteins in the developing mouse cortex/hippocampus. (a) Representative immunoblotting of full-length AR (AR110) and smaller isoform (AR70) in the female and male cortex/hippocampus on postnatal days 0 (PN0) and 21 (PN21). (b) Relative levels of AR110 and AR70 in the female (top panels) and male (bottom panels) mouse cortex/hippocampus. AR protein levels were quantified as relative to average of neonatal mice (Day 0) of the same sexes. ^*^Indicates significant difference from neonatal expression by *t*-test (*P* < 0.05). Bars are mean ± SEM. PN, postnatal day.

Since AR mRNA expression increased with age, we next measured AR protein levels in the mouse cortex/hippocampus collected on PN0 and PN21 to determine if there was an age-dependent increase in AR proteins. All PN0 and PN21 male or female samples were run on single blots (Figure 4(a)), and the relative AR levels in individual samples were normalized to the mean level of PN0 mice of the same sex. Regardless of sex, expression of the 110-kDa AR protein in the cortex/hippocampus was higher on PN21 than PN0 in both males (PN0, 1.00 ± 0.04, *n* = 6 versus PN21, 8.45 ± 0.74, *n* = 7) (*P* < 0.001) and females (PN0, 1.00 ± 0.04, *n* = 6 versus PN21, 5.88 ± 0.34, *n* = 7) (*P* < 0.001) (Figure 4(b), left panels). Similarly, relative protein levels of the 70-kDa AR isoform also increased as age advanced in both males and females (*P* < 0.001) (Figure 4(b), right panels).

**Figure 5:**
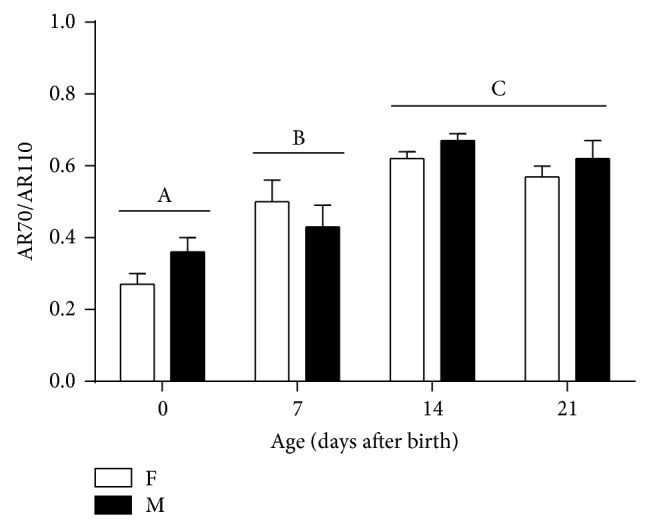
Effect of age and sex on the ratios of the smaller AR isoform (AR70) to full-length AR (AR110) in the developing female (F) and male (M) mouse cortex/hippocampus. Different letters indicate significant differences between two ages based on post hoc Tukey's test (*P* < 0.05). Bars are mean ± SEM.

We found no sex difference in the ratios of the smaller AR isoform to the full-length AR, but there was an age-dependent increase in the ratios: 32.0 ± 2.9% (PN0), 44.9 ± 4.3% (PN7), 64.7 ± 1.8% (PN14), and 59.5 ± 3.1% (PN21) (*P* < 0.001) (Figure 5).

## 4. Discussion

Our study reveals that, as age advanced, levels of AR mRNA and two protein isoforms, 110 kDa (full-length) and 70 kDa, increase in the male and female mouse cortex/hippocampus during the first three weeks after birth (Figures 1(a) and 4). To our knowledge, this investigation represents the first report of identification of a smaller AR isoform in the developing mouse brain. In addition, we have observed a novel timeline when sexually dimorphic AR expression at the protein, not mRNA, level occurs in the developing mouse cortex/hippocampus, with males showing about 39% more full-length AR than females on PN7 (Figure 3(b)). The sex-specific development of rodent cerebral cortex and hippocampus are sensitive to sex steroids. For example, adult male mice possess a thicker cerebral cortex than females, which depends on gonadal hormones, rather than the complement of sex chromosomes [19]. Similar to the cortex, the hippocampus of male rats has larger CA3 pyramidal cell field volumes and soma sizes than females, which can be reversed by prenatal treatment of male rats with antiandrogen flutamide or females with testosterone propionate (TP) and dihydrotestosterone (DHT) [42]. The presence of AR in the developing mouse cerebral cortex and hippocampus may serve as an important mechanism through which androgens can directly regulate the development of dimorphic structures in these two brain regions underlying behavioral and functional differences between the sexes.

Given sexual dimorphism in AR protein expression in the mouse brain, the explanation could be as simple as regulation in AR protein synthesis (translation) and/or stability (degradation) between the sexes via ligand-receptor interaction. While gonadectomy causes a loss of AR protein, androgen supplement after castration restores AR levels in the adult mouse brain and rat prostate [27, 43]. In vitro studies have further demonstrated that the ligand-receptor interaction stabilizes AR protein from degradation and possibly increases its synthesis to upregulate AR expression [44–47]. Thus, the perinatal rise in circulating T released by the developing testes may exploit similar molecular and cellular mechanisms to increase AR protein levels in the male cortex/hippocampus on PN7 (Figure 3(b)).

In addition, masculine elevation of cortical and hippocampal AR protein might be simply caused by increasing the number of AR-expressing cells located in these brain regions via neurogenesis, migration, and differentiation. In good agreement with masculine expression of cortical and hippocampal AR protein shown in Figure 3(b), Nuñez et al. found greater AR-immunoreactive cell density in the primary visual cortex of male rats than females on PN10, not at younger ages, and further demonstrated that such a sex difference in AR expression was laminar-specific (limited to layers II–IV) [48]. The latter suggests that more cortical neurons are likely to migrate into the superficial layers of the male cortex, where they differentiate and express AR. We speculate that, similar to the rat visual cortex, increasing the number of AR-expressing cells via migration might be responsible for the elevated masculine levels of cortical/hippocampal AR protein in PN7 mice (Figure 3(b)). Consistent with their immunocytochemical data, Nuñez and colleagues also reported higher AR mRNA levels detected in the male visual cortex than in females by in situ hybridization [48] whereas we found no sex difference in AR mRNA in the developing mouse cortex/hippocampus (Figure 1(a)). Besides differences in species (rat versus mouse) and quantification techniques (i.e., in situ hybridization versus RT-qPCR), these discrepancies may be due to the heterogeneity of the cortical and hippocampal neurons, which may mask small variations in AR mRNA levels occurring in subpopulations of cells when they are mixed with the rest of the cortex and hippocampus.

Other than the cortex and hippocampus, sexual dimorphism in AR expression has also been noted in the rodent BNST and mPOA, which appears near the first postnatal week and then persists throughout adulthood [26–29]. During late gestation and on the day of birth, there is a transient rise in circulating T in male mice, which is critical for masculine development of AR expression in these two brain regions. Juntti et al. found that treatment of neonatal female pups with TP increased the number of AR-positive cells in their BNST and mPOA on PN6 [29]. Similar to mice, neonatal gonadectomy lowered levels of AR mRNA in these two brain regions of PN10 male rats, and replacement of TP to gonadectomized animals maintained their AR mRNA content at levels equal to those in intact males [49]. It has been further demonstrated that estrogenic metabolites aromatized from testosterone may act via ERs to regulate sex differences in AR expression in the BNST and mPOA. This conclusion is based on the findings that estradiol benzoate (EB) masculinizes AR expression in females while demasculinization of AR expression is found in males lacking functional aromatase [29]. Since there is robust ER*α* expression in the developing mouse cortex during first week after birth, the perinatal rise in T might be converted into estradiol locally to activate ER*α* to similarly drive increased AR protein expression in the male mouse cortex and hippocampus during early postnatal development. Unlike the BNST and mPOA, sexually dimorphic AR protein expression in the developing mouse cortex/hippocampus disappears by PN14 (Figure 3(b)). While two studies reported higher AR mRNA expressed in the cortex of male mice than in females [50, 51], Thakur et al. and our data shown in Figure 2 show that the full-length AR protein levels seem to remain sexually indifferent in the adult mouse cortex and hippocampus [50]. These results indicate that AR transcription and translation as well as posttranscriptional and posttranslational modifications in the developing cortex and hippocampus might be regulated differently as compared to the BNST and mPOA. In addition, several unique features render AR cells in the cortex distinctly different those in the hypothalamus. First, in comparison to AR-immunoreactive cells in the hypothalamus, the intensity of AR label per cell in the cerebral cortex is moderate [52]. Second, the total population of AR-positive cells is greater in the telencephalon, including the cortex and hippocampus, than in the diencephalon. Third, immunoreactivity for intracellular AR is abundant within the layers II/III and V/VI of the sensory and motor cortex, in which the majority of AR-positive cells are pyramidal neurons [53]. Fourth, ARs in the cerebral cortex are located not only in the nucleus but also in the axons and dendrites while AR expression in the hypothalamic regions is exclusively nuclear [54]. Although sexual dimorphism in AR expression in the mouse cortex/hippocampus does not persist beyond PN7 or into adulthood, differences in circulating androgens between the sexes can still modulate AR function in a sex-dependent manner.

In mice, AR transcripts can be detected in the neuroepithelium of brain vesicles as early as E11 and later appear in the cerebral cortex and hippocampus as well as the hypothalamus during E15-E16 [55]. In contrast to a relatively small, transient difference in AR expression between the sexes on PN7 (39% more in males than in females), we demonstrate a robust, age-dependent elevation of AR mRNA and proteins in the male and female mouse cortex/hippocampus during early postnatal development. When comparing PN0 and PN21 mice, there are a 59- and an 11-fold increases in mRNA and protein levels, respectively (Figures 1(a) and 4(b)). The difference in the fold changes between AR mRNA and protein in the cortex/hippocampus suggest differential rates of AR transcription and translation as well as degradation of its mRNA and protein. In addition, the differences in detection sensitivity and quantification mechanisms between RT-qPCR and immunoblotting might also be part of the cause.

In addition to differential regulation of transcription, translation, and degradation, developmental changes in cortical and hippocampal AR protein levels in the developing brain might be the result of increased migration, proliferation, and differentiation of AR-expressing cells with age, which is supported by previous studies. Tripathi et al. found moderate numbers of AR-immunopositive cells in the CA1, CA2, and subiculum at PN0, and then the numbers of AR-positive cells in these three regions increased at PN15 and reached the maximum by PN45 [56]. Similar to the hippocampus, the rat primary visual cortex and anterior cingulate/frontal cortex both show an increase in AR-immunoreactive cell density with age, lower on PN0 than on PN10 [48]. Additionally, AR cells were observed only in layers V and VI of the neonatal visual cortex. As age advances, AR immunoreactivity was observed in more superficial layers, layers II–VI, on PN4, followed by robust increases in the AR cell density in layers II–IV and modest increases in layers V and VI on PN10. These findings indicate that the developmental changes in the density and number of AR-immunoreactive cells in the cortex and hippocampus are region-specific, which might result from neuronal migration and differentiation occurring in the specific layers of the cerebral cortex.

In the present study, we observed a smaller AR isoform with a molecular weight of approximately 70 kDa coexpressed with the full-length AR (~110 kDa) in the developing and adult mouse cortex/hippocampus and hypothalamus (Figures 2, 3(a), and 4(a)). Both AR proteins are recognized by the same antibody raised against a peptide mapping at the aminoterminus of human AR, suggesting that the smaller AR might be composed of at least part of the aminoterminal sequence of the full-length AR. A variety of smaller AR isoforms have been previously identified and characterized in prostate cancer cells [57]. Like the mouse cortex/hippocampus, 22Rv1 cells, a human prostate carcinoma epithelial cell line, are also found to express two AR protein species of ~112 and 75–80 kDa [58]. Two mechanisms have been shown to produce the truncated AR protein species. First, it can be produced via proteolytic degradation of full-length AR by calpain-2 [59] or caspase-3 [60]. Second, alternative splicing could be another important contributor to the synthesis of truncated AR species lacking the AR ligand binding domain (LBD) [61]. Using antibody mapping, the smaller AR species is composed of the AR aminoterminal domain and DNA binding domain but lacks the carboxyl-terminal LBD [58]. Further, the truncated AR is nuclear, binds to ARE, and regulates gene transcription independent of androgens but does not interact with full-length AR. Selective abolition of expression of the full-length AR or both ARs by siRNA demonstrates that androgen-independent AR target gene expression and cell growth are mediated by the smaller AR species. Although the exact function of the 70-kDa AR isoform in the brain is unclear, based on the in vitro data, we speculate that this smaller AR isoform might regulate the transcription of specific genes responsible for cortical and hippocampal function via interfering with T-dependent and T-independent pathways.

## 5. Conclusion

In summary, AR mRNA and proteins are present in the developing mouse cortex/hippocampus on the day of birth in both males and females, followed by a robust increase in AR expression at the mRNA and protein levels with age during the early postnatal period. This developmental profile of AR expression indicates that age-dependent regulation of AR transcription, splicing, and/or translation might be involved in programming the structure of the cortex and hippocampus, ultimately contributing to the control of social and cognitive behaviors as well as the underlying neural circuits. Although the sexually differentiated expression of full-length AR protein in the developing mouse cortex/hippocampus is transient, whenever AR protein expression is similar between the sexes, the differences in circulating androgens could still play an important role in modulating AR function in a sex-dependent fashion. Future studies will focus on investigating AR expression specifically in discrete subregions of the cortex and hippocampus.

## Figures and Tables

**Table 1:** List of oligonucleotide primers used for RT-PCR and RT-qPCR.

Gene symbol	RefSeq accession	Forward primer (5′-3′)	Reverse primer (5′-3′)	Product size (bp)	Annealing temperature (°C)
*Actb* ^*^	NM_007393.3	CCAGATCATGTTTGAGACCTTCAA	CCAGAGGCATACAGGGACAGC	78	59
*Ar *	NM_013476.3	AGAATCCCACATCCTGCTCAA	AAGTCCACGCTCACCATATGG	133	59
*Rpl13a* ^*^	NM_009438.4	ATGAGGTCGGGTGGAAGTACC	CAGGAGTCCGTTGGTCTTGAG	179	62
*Xist *	NR_001570.2	GCTGGTTCGTCTATCTTGTGGG	GGATCCTGCACTGGATGAGT	225	58

^*^Indicates a reference/housekeeping gene.

**Table 2:** Body weights of the male and female mice at different ages used in the current study as well as the tissue weights of their cortex/hippocampus.

Age (days after birth)	Sex	*N*	Body weight (g)	Tissue weight (mg)
0	Female	26	1.26 ± 0.02	24.63 ± 1.29
	Male	23	1.24 ± 0.03	26.58 ± 1.49

7	Female	14	3.99 ± 0.14	79.11 ± 4.77
	Male	16	3.66 ± 0.12	86.67 ± 4.78

14	Female	14	7.50 ± 0.28	114.21 ± 7.98
	Male	15	7.54 ± 0.24	108.29 ± 4.72

21	Female	13	8.15 ± 0.42	115.86 ± 5.97
	Male	16	8.79 ± 0.18	119.44 ± 3.92

Data are shown as mean ± SEM. *N*, number of mice.

**Table 3:** Relative protein levels of the smaller androgen receptor (AR) isoform (AR70) in the male and female mouse cortex/hippocampus.

Age (days after birth)	Sex	*N*	AR70 levels (versus females of the same age)
0	Female	6	1.00 ± 0. 21
	Male	7	0.81 ± 0.09

7	Female	6	1.00 ± 0.12
	Male	7	1.09 ± 0.12

14	Female	6	1.00 ± 0.06
	Male	7	1.08 ± 0.15

21	Female	5	1.00 ± 0. 21
	Male	6	0.94 ± 0.17

Data are shown as mean ± SEM. *N*, number of mice.
